# Case report: Remarkable response to later-line surufatinib in an adult patient with liver metastatic of pancreatoblastoma

**DOI:** 10.3389/fphar.2024.1361628

**Published:** 2024-06-14

**Authors:** Qingqing Leng, Wanrui Lv, Heqi Yang, Xiaofen Li, Weiya Wang, Ke Cheng, Chen Chang, Dan Cao

**Affiliations:** ^1^ Division of Abdominal Tumor Multimodality Treatment, Cancer Center, West China Hospital, Sichuan University, Chengdu, Sichuan, China; ^2^ Department of Oncology, Meishan Municipal People’s Hospital, Meishan, Sichuan, China; ^3^ Department of Pathology, West China Hospital, Sichuan University, Chengdu, Sichuan, China

**Keywords:** pancreatoblastoma, adult patient, liver metastasis, surufatinib, disitamabvedotin

## Abstract

Pancreatoblastoma (PB), a neoplasm derived from pancreatic follicular cells, primarily affects the pediatric population. Although infrequent in adults, it is associated with a considerably worse prognosis. Approximately one-third of patients are diagnosed with metastatic disease, with liver metastases being the most prevalent. Diagnosis relies on histopathological alterations including squamous vesicles, positive staining for CK8/CK18/CK19, and nuclear displacement of β-catenin. Additionally, liver metastases demonstrate substantial enhancement during the arterial phase of a contrast-enhanced computed tomography (CT) scan. Surgical resection serves as the principal therapeutic approach for addressing primary lesions and liver metastatic PB. In instances where surgical intervention is not viable, patients may derive benefits from systemic therapy and radiotherapy. This particular case report presents the clinical details of a 27-year-old female patient diagnosed with PB, who subsequently developed multiple liver metastases following a pancreaticoduodenectomy. Genomic examinations revealed the presence of ERBB2 amplification, RAD54L deletion, low TMB-L, and MSS in the patient. Despite the patient undergoing chemotherapy and Her-2 targeted therapy in conjunction with immunotherapy, no reduction in lesion size was observed until the administration of surufatinib. Subsequently, a notable outcome ensued, where the metastatic lesions were effectively excised via surgical intervention. Surufatinib has demonstrated a progression-free survival (PFS) of no less than 14 months, and the patient’s survival has endured for a duration of 33 months. This indicates the potential efficacy of surufatinib as a viable therapeutic alternative for adult patients afflicted with PB.

## Introduction

Pancreatoblastoma (PB) is a malignant tumor that originates in the pancreas and is composed of differentiated cells and squamous cell nests (Omiyale, 2021). It was first identified by Becker in 1975 and named PB by Kissan. PB primarily affects children aged 1–8 years old and is rare in adults, accounting for less than 1% of all pancreatic tumors (Liu et al., 2022). Prognosis tends to be worse for adult patients compared to pediatric patients (Zhang et al., 2020). Due to its low incidence, the exact causes and molecular pathogenesis of PB remain unknown. Symptoms include abdominal pain, diarrhea, an abdominal mass, jaundice, and progressive weight loss. The liver is the most common site of metastasis in adult PB. On a contrast-enhanced CT scan, liver metastases significantly enhance during the arterial phase. Histopathological examination is vital for diagnosis and differentiating PB from solid pseudopapillary tumors and adenoid cell carcinoma of the pancreas (Salman et al., 2013). Salman’s literature review reported a median overall survival of 15 months for adults with PB (Huang et al., 2019). Complete removal of both primary and metastatic tumors is the preferred treatment for PB. In cases where the tumor has infiltrated major blood vessels or other organs, platinum-based chemotherapy regimens may be considered. Some studies have shown that the prognosis is poor in locally advanced or metastatic cases. There is limited data on the standard clinical management of recurrent and/or metastatic PB (Dall’igna et al., 2010; Argon et al., 2017; Dhamne and Herzog, 2015).

Herein, we report a case of an adult with liver metastatic PB who developed liver metastases post-surgery. However, the administration of oral surufatinib resulted in a significant reduction in liver metastases, suggesting that surufatinib could be a promising treatment option for patients with PB.

## Case description

**FIGURE 1:**
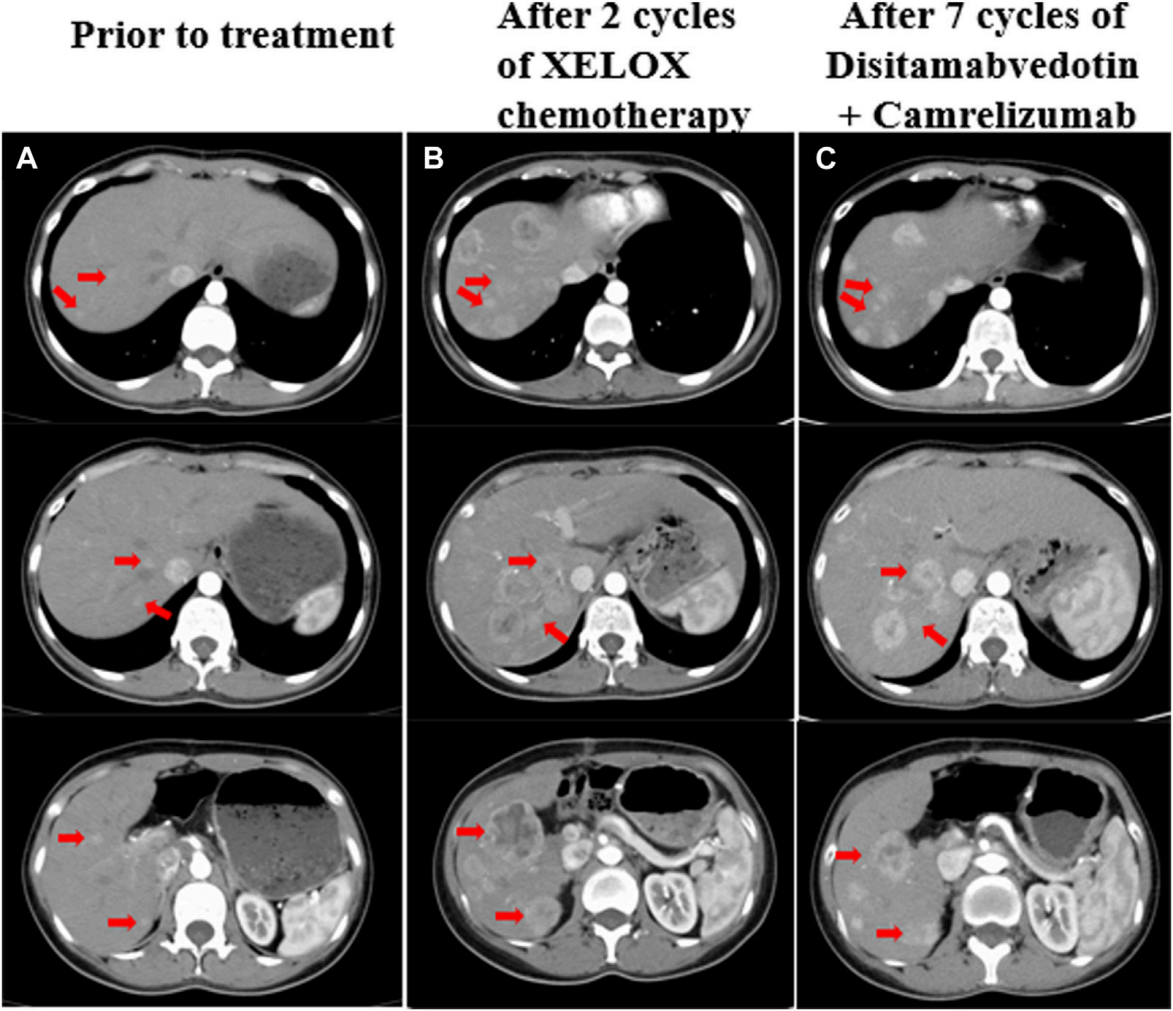
**(A)** Liver metastases prior to treatment, a CT scan showed multiple slightly hypodense nodules in the hepatic parenchyma with blurred margins and uneven mild enhancement, the largest of which was about 1.2 cm. **(B)** CT revealed increased and enlarged liver metastasis despite two cycles of chemotherapy, the largest one was about 4.8 cm*4.1 cm. **(C)** CT showed that the liver metastases were slightly smaller than before after treatment with disitamabvedotin in combination with camrelizumab. The red arrows on the image illustrate the changes in liver metastases that were observed both before and during treatment.

**FIGURE 2:**
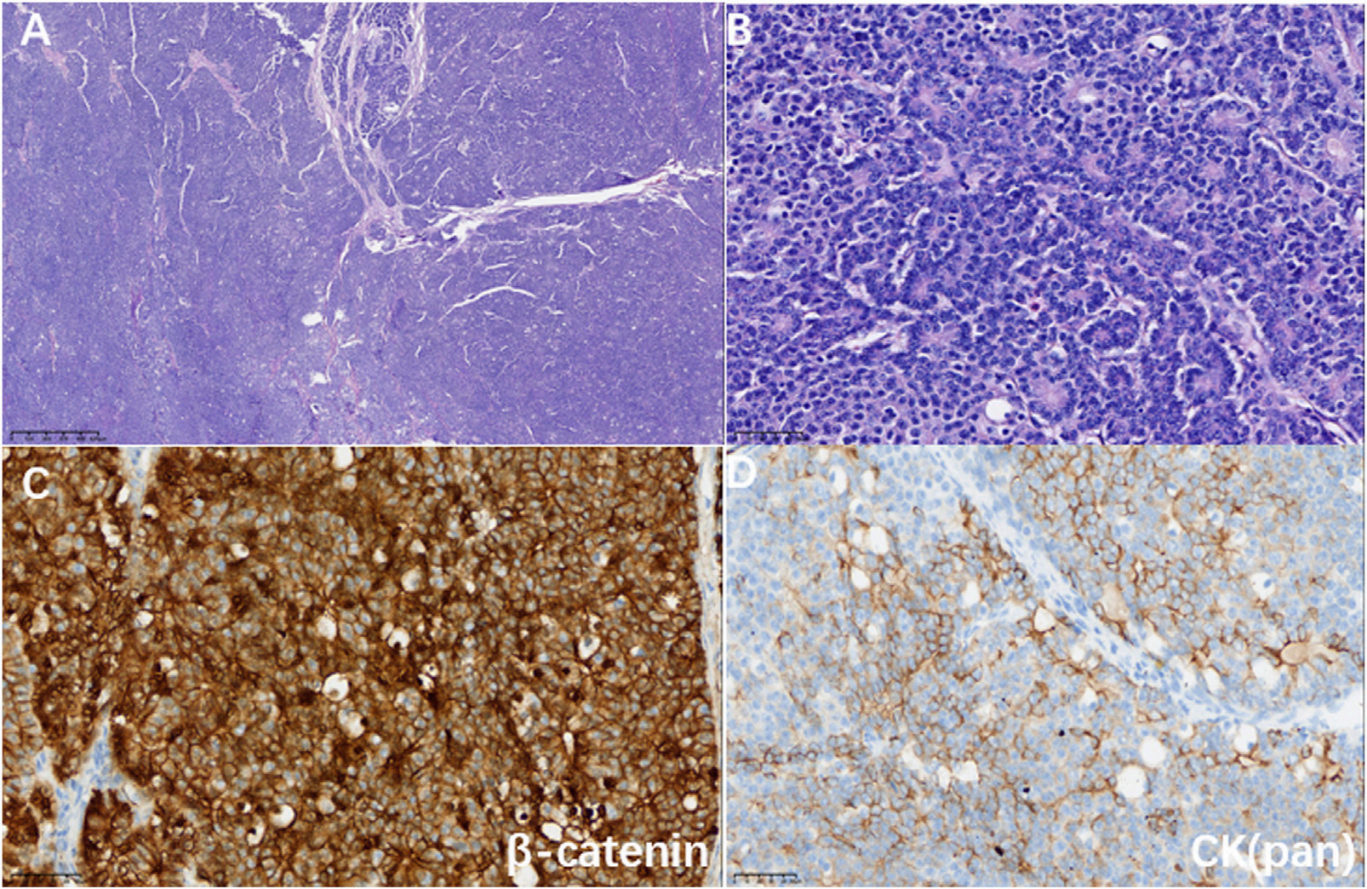
**(A)** Hematoxylin-eosin staining showed a map-like tumor (magnification 40×). **(B)** Tumor cells were arranged in an organoid pattern around small lumens with mildly heterogeneous nuclei and granular eosinophilic cytoplasm (magnification ×400). **(C)** Immunohistochemistry showed a positive nuclear and cytoplasm marker for β-catenin (magnification ×400); **(D)** Immunohistochemistry showed a partially positive marker for CK (pan) (magnification ×400).

A 27-year-old female with no physical complaints was incidentally found to have a pancreatic lesion during a routine abdominal ultrasound color Doppler examination. Subsequent abdominal contrast-enhanced CT imaging revealed the presence of a pancreatic tumor as the most likely diagnosis. In January 2021, the patient underwent a pancreaticoduodenectomy in another hospital. Postoperative pathology indicated PB, with no metastasis in six regional lymph nodes and clear resection margins. Following the surgery, the patient was regularly monitored without receiving adjuvant treatment. In September 2021, a follow-up abdominal CT showed multiple slightly hypodense nodules in the hepatic parenchyma, with blurred margins and uneven mild enhancement, the largest being approximately 1.2 cm (Figure 1A). A CT-guided percutaneous liver biopsy revealed multiple solid and squamous cell clusters infiltratively distributed in the liver tissue. The biopsy also showed intravascular tumor thrombus formation and keratinized material aggregation in the focal area. Immunohistochemical staining results: CK(Pan) (partially +), p63, CgA, and Syn all (individually +), Ki-67(MIB-1) (+, ∼5%), Beta-Catenin (+, membrane + nucleus), E-cadherin (+), CD10 (+), GPC3 (weakly +). The diagnosis was that there were metastases of PB to the liver (Figure 2). Genetic testing identified multiple mutations, including amplification of ERBB2 with a mutation frequency of 3.6, FLCN deletion, MLL2 variant, TMB-L, MSS, and RAD54L deletion.

Since the rapid development of multiple liver metastases post-surgery, surgical intervention was not feasible. In December 2021, the patient began first-line chemotherapy, which was XELOX (oxaliplatin 130 mg/m2 on day 1 + capecitabine 1,000 mg/m2 twice daily from day 1 to day 14, repeated every 3 weeks). The patient tolerated chemotherapy very well, but after two cycles, the CT scan revealed a significant increase in size and number of liver metastases. The largest metastasis measured approximately 4.8 * 4.1 cm, indicating disease progression according to the Response Evaluation Criteria in Solid Tumors version 1.1 (RECIST 1.1) (Figure 1B). Considering the ERBB2 amplification of the tumor and the lack of a standard treatment for refractory PB, a second-line treatment of Herceptin anti-ERBB2 with chemotherapy was recommended. However, the patient declined this option. After consulting with the patient, experts from a multidisciplinary team suggested a combination treatment involving an anti-ERBB2 targeted drug, an immune checkpoint inhibitor, and TAE (Transarterial embolization) for liver metastases. In January 2022, the patient began second-line therapy with the anti-ERBB2 drug disitamabvedotin and the immune checkpoint inhibitor camrelizumab (disitamabvedotin 100 mg on day 1 + camrelizumab 200 mg on day 1, repeated every 3 weeks). During treatment with this regimen, the patient experienced adverse effects such as fever, skin erythema, and weight loss. Therefore, the dose was adjusted based on the patient’s weight change and adverse effects. Transarterial embolization (TAE) was performed on the liver lesions in March 2022. During 7 months of second-line treatment, CT scans were performed every 2 months and evaluated as stable disease (SD) based on RECIST 1.1 (Figure 1C).

**FIGURE 3:**
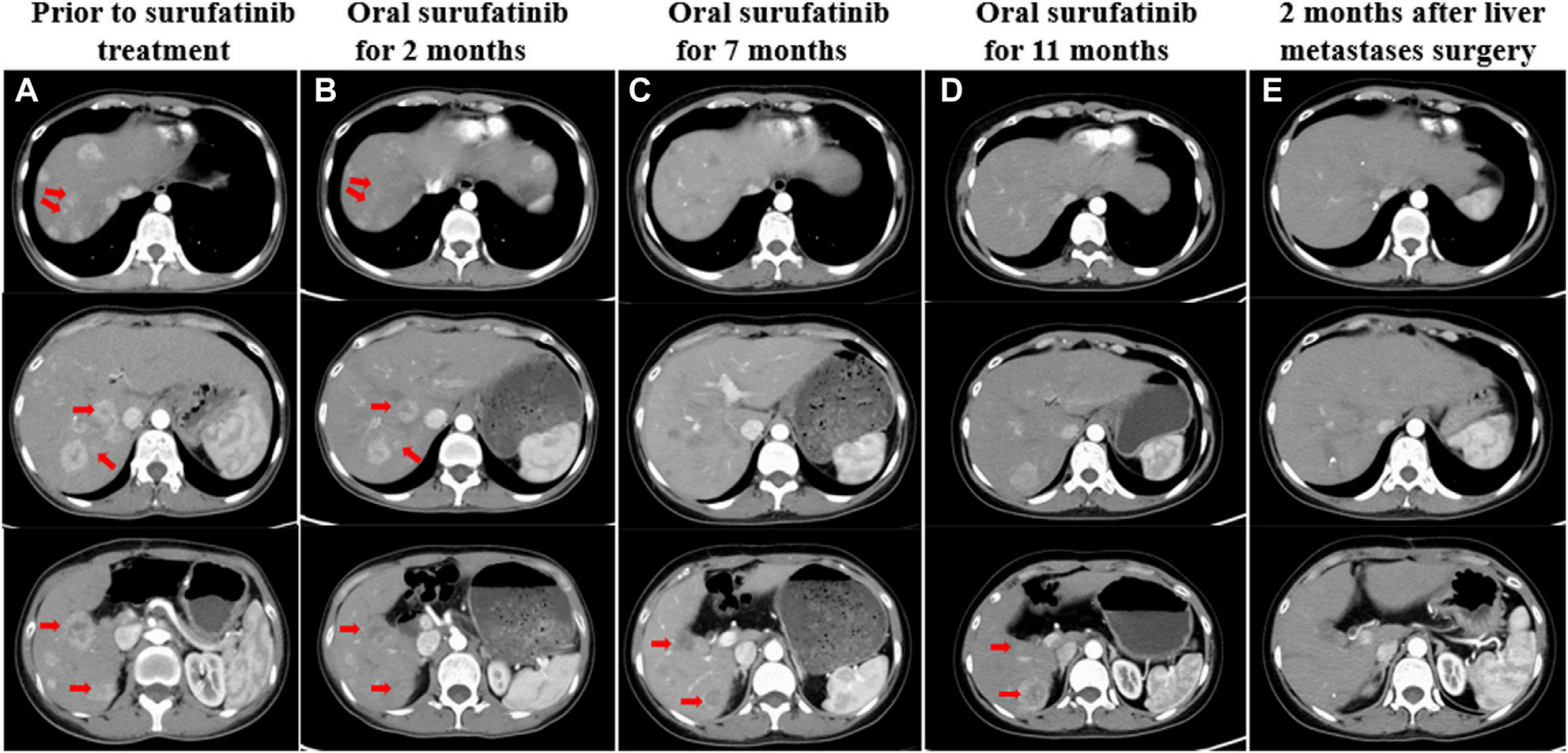
**(A)** CT showed liver metastases before treatment with surufatinib. **(B)** Two months after taking surufatinib, CT showed that some of the liver lesions were reduced in size, with decreased enhancement and obvious liquefied necrosis. **(C)** After 7 months of surufatinib administration, CT showed liver metastases with clearer borders, reduced enhancement, and reduced extent than before. **(D)** After taking surufatinib for 11 months, CT revealed that some of the liver metastases were slightly enlarged and some were slightly reduced. **(E)** After surgical resection of liver metastases, the patient continued to take surufatinib, and 2 months later, CT showed that most of the hepatic metastases intensified significantly less than before, and the morphology became irregular, and there was still enhancement within some of the lesions. The red arrows on the image illustrate the changes in liver metastases that were observed both before and during treatment.

**FIGURE 4:**
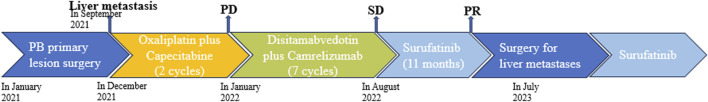
Schematic diagram of the antitumor treatment process. The patient had surgery to remove the primary tumor caused by PB but developed liver metastasis. Chemotherapy was ineffective, so they received disitamabvedotin and camrelizumab, which were able to control the growth of metastasis, but slowly. Targeted therapy with surufatinib gradually reduced the size of the metastasis. After 11 months, the patient underwent surgery to remove the metastasis and continued taking surufatinib as post-surgery treatment.

As the CT scan showed significant enhancement of liver lesions in the arterial phase, indicating a rich blood supply, the treatment was changed to a daily oral anti-angiogenic drug called surufatinib in August 2022 (Figure 3A). Since the patient only weighed 30 kg at the time, a reduced dose of 150 mg was administered. Until February 2023, abdominal CT scans revealed that the liver metastases had shrunk by nearly 45% compared to pre-surufatinib measurements (Figures 3B, C). Consequently, the patient obtained a partial response (PR) based on RECIST 1.1, which was maintained from February 2023 to June 2023. In July 2023, the dose of surufatinib was increased to 200 mg daily due to slight enlargement of some liver metastases and reduction in others (Figure 3D). Moreover, following a multidisciplinary consultation, liver metastasis resection was planned, motivated by tumor shrinkage and the patient’s request for surgery. On 18 July 2023, the patient underwent surgical resection and microwave ablation of liver metastasis. Postoperative pathology confirmed PB. Postoperative CT scans showed reduced enhancement in most liver metastases, although some lesions continued to enhance (Figure 3E). After the surgery, the patient continued taking daily oral surufatinib (200 mg), achieving a progression-free survival (PFS) of at least 14 months up to now. During surufatinib treatment, no serious adverse events were noted, except for grade 1 fatigue and nausea, which were symptomatically treated and resolved. Currently, the patient has been alive for 33 months since the diagnosis of PB (Figure 4).

## Discussion

PB is a rare malignant neoplasm that predominantly affects pediatric patients and is less commonly observed in adults. Common clinical manifestations of PB include abdominal pain and diarrhea. In contrast, our adult female patient did not present any such symptoms. Instead, pancreatic occupancy was discovered during a physical examination, and the patient underwent a pancreaticoduodenectomy. Postoperatively, the patient did not receive postoperative adjuvant therapy. Eight months after surgery, multiple liver metastases, which were visibly enhanced in the arterial phase of CT scans, were discovered.

The rarity of PB cases has led to a lack of established treatment guidelines. However, a comprehensive analysis of previously documented data supports the rationale for employing oxaliplatin-based chemotherapy as a viable therapeutic approach in advanced PB among adult patients (Berger et al., 2020). Therefore, we opted for first-line treatment consisting of oxaliplatin combined with capecitabine chemotherapy.

Regrettably, the patient experienced disease progression after two cycles of chemotherapy. Due to the limited literature on second-line and salvage treatment of recurrent metastatic PB, new therapeutic strategies must be explored in adults. Although a single case study reported no recurrence within 51 months after autologous peripheral blood hematopoietic, it also highlighted serious adverse effects (Elghawy et al., 2021). Considering the patient’s genetic test indicating ERBB2 amplification, chemotherapy combined with anti-ERBB2 targeted therapy was considered as an alternative treatment option. However, the patient declined chemotherapy and expressed a strong preference for surgical intervention. After a multidisciplinary consultation, the decision was made to use targeted therapy combined with immunotherapy as a second-line treatment option. Disitamabvedotin (RC48), the third HER2-targeted antibody drug coupling agent (ADC), has been approved by the National Drug Administration (NMPA) of China in 2021 for locally advanced or metastatic gastric or gastroesophageal junction cancer (Yu et al., 2022). However, combining disitamabvedotin with the PD-1 inhibitor camrelizumab resulted in stable disease (SD) only. We speculated that the patient’s low Her-2 expression (Her-2 1+) may have contributed to this outcome. In response to the patient’s persistent desire for surgery, adjustments were made to their treatment regimen.

Surufatinib, a novel oral small-molecule tyrosine kinase inhibitor, effectively targets multiple receptors, including VEGFR1, VEGFR2, VEGFR3, FGFR1, and CSF-1R. Its unique mechanism of action involves the simultaneous inhibition of angiogenesis (VEGFR and FGFR1) and tumor immune evasion (CSF-1R), potentially leading to enhanced antitumor activity (Xu, 2021). In the SANET-p and SANET-ep Phase III clinical trials, surufatinib significantly extended PFS in patients with pancreatic and extra-pancreatic tumors compared to placebo. Common adverse events from surufatinib were hypertension, proteinuria, and diarrhea, which are typical for small-molecule anti-angiogenic TKIs. While more patients on surufatinib had dose interruptions or reductions than those on placebo, most tolerated the adverse events well (Xu et al., 2020). Surufatinib was chosen in this case due to the significant arterial enhancement of liver metastases on CT scans, indicating its rich blood supply. In addition, our decision was influenced by the drug’s efficacy in treating pancreatic neuroendocrine tumors. Following surufatinib treatment, the patient with liver metastasis from PB showed ongoing tumor reduction, leading to eventual surgical intervention. During surufatinib treatment, the patient experienced only grade 1 adverse events such as fatigue and nausea, which resolved with symptomatic treatment. The patient has survived for 33 months since diagnosis, surpassing the median 18-month survival for adult PB patients (Li et al., 2021). This case suggests that surufatinib may benefit patients with metastatic PB.

## Conclusion

Adult PB with multiple liver metastases is rare. These metastases are characterized by a rich blood supply. The absence of effective targeted therapies has led to the investigation of surufatinib as a potential treatment option. To our knowledge, this is the first reported case of adult PB with liver metastases treated with surufatinib, and the patient has survived for more than 33 months. This case suggests the potential of surufatinib as a promising treatment for PB with liver metastases. However, further confirmation through large sample studies is necessary.

## Data Availability

The original contributions presented in the study are included in the article/Supplementary material, further inquiries can be directed to the corresponding author.
